# Dispersal of Engineered Male *Aedes aegypti* Mosquitoes

**DOI:** 10.1371/journal.pntd.0004156

**Published:** 2015-11-10

**Authors:** Peter Winskill, Danilo O. Carvalho, Margareth L. Capurro, Luke Alphey, Christl A. Donnelly, Andrew R. McKemey

**Affiliations:** 1 Medical Research Council Centre for Outbreak Analysis and Modelling, Department of Infectious Disease Epidemiology, School of Public Health, Faculty of Medicine, Imperial College London, St Mary's Campus, London, United Kingdom; 2 Oxitec Limited, Oxford, United Kingdom; 3 Instituto Nacional de Ciência e Tecnologia em Entomologia Molecular (INCT-EM), Universidade Federal do Rio de Janeiro, Ilha do Fundão, Rio de Janeiro, Brazil; 4 Departamento de Parasitologia, Universidade de São Paulo, São Paulo, Brazil; 5 Instituto Nacional de Ciência e Tecnologia em Entomologia Molecular (INCT-EM), Rio de Janeiro, Brazil; 6 The Pirbright Institute, Ash Road, Pirbright, Woking, United Kingdom; 7 Department of Zoology, University of Oxford, Oxford, United Kingdom; North Carolina State University, UNITED STATES

## Abstract

**Background:**

*Aedes aegypti*, the principal vector of dengue fever, have been genetically engineered for use in a sterile insect control programme. To improve our understanding of the dispersal ecology of mosquitoes and to inform appropriate release strategies of ‘genetically sterile’ male *Aedes aegypti* detailed knowledge of the dispersal ability of the released insects is needed.

**Methodology/Principal Findings:**

The dispersal ability of released ‘genetically sterile’ male *Aedes aegypti* at a field site in Brazil has been estimated. Dispersal kernels embedded within a generalized linear model framework were used to analyse data collected from three large scale mark release recapture studies. The methodology has been applied to previously published dispersal data to compare the dispersal ability of ‘genetically sterile’ male *Aedes aegypti* in contrasting environments. We parameterised dispersal kernels and estimated the mean distance travelled for insects in Brazil: 52.8m (95% CI: 49.9m, 56.8m) and Malaysia: 58.0m (95% CI: 51.1m, 71.0m).

**Conclusions/Significance:**

Our results provide specific, detailed estimates of the dispersal characteristics of released ‘genetically sterile’ male *Aedes aegypti* in the field. The comparative analysis indicates that despite differing environments and recapture rates, key features of the insects’ dispersal kernels are conserved across the two studies. The results can be used to inform both risk assessments and release programmes using ‘genetically sterile’ male *Aedes aegypti*.

## Author Summary

Vector control using releases of sterile insects is a well-known approach. ‘Genetically sterile’ male *Aedes aegypti* have been developed and released in a modern realisation of the sterile insect technique. Released engineered males seek out and mate with wild females, with the resultant offspring dying before they reach maturity. Control of a wild vector population can therefore be achieved by maintaining sustained releases of sterile males whilst ensuring sufficient distribution and coverage of released males across the target area. In order to efficiently plan releases of these, individuals’ detailed knowledge of how they disperse in the field is required. We present an analysis of the dispersal of these engineered male *Aedes aegypti* using data from field experiments in Brazil. Our results provide detailed information on how the mosquitoes disperse over their potential flight range.

## Introduction

Dengue, an arbovirus, has seen recent re-emergence and spread on a global scale [1] and is now responsible for an estimated 390 million infections annually [2]. The vector of dengue is the *Aedes* mosquito, with *Ae*. *aegypti* and *Ae*. *albopictus* responsible for the majority of disease transmission [3]. The release of ‘genetically sterile’ male *Aedes* mosquitoes has been demonstrated to be a valuable additional tool by which the vector can be controlled [4]. Understanding the ability of the released ‘genetically sterile’ insects to disperse, and their behaviour whilst doing so, is an important step in designing robust, efficient and effective releases. Attaining adequate coverage of released sterile insects across a given area is a major operational challenge of a sterile insect control effort [4]. Knowledge of the distribution of dispersal distances of released insects will improve our ability to target releases, obtain required coverage densities, confidently predict the potential spatial range of a release and is a key element for the assessment of risk.

Independently conceived by Petersen in 1896 and Lincoln in 1930 [5], mark-recapture, capture-recapture or mark-release-recapture studies (hereafter referred to as MRR) have since become a key set of ecological methods. MRR allows inference to be drawn about a number of important ecological factors including the estimation of population size and quantification of dispersal and survival. The methods have been used across a diverse range of species, from whales [6] to fruit flies [7], and have seen extensive use in mosquito ecological studies.

Analysis of the location of recaptured marked insects with respect to the release point allows inference to be made about the dispersal of the released insects. A number of MRR studies have been performed with the aim of assessing the dispersal ability of both lab [8–11] and ‘genetically sterile’ [12] strains of male *Ae*. *aegypti*. However, these studies often document only the mean distance travelled (MDT) [8–12] or range [11,12] of dispersal of the released insects. Common measures of range are the flight range 50% and flight range 90% (FR50 and FR90 respectively) which are estimates of the distance within which 50% or 90% of all insects are expected to disperse [12,13]. The MDT and flight range are intuitive summary measures but do not characterise dispersal well when the distribution of dispersal distances is positively skewed with a long tail.

For greater insight, a better understanding of the distribution of dispersal distances can be obtained by incorporating dispersal kernel theory, popular in studies of population spread [14] and seed dispersal [15], into a generalised linear model (GLM) framework [16,17]. Dispersal kernels represent the distribution of dispersal distances over the whole flight range. They can take a wide range of forms with the flexibility to represent dispersal for a diverse range of species [18].

This study attempts improve the characterisation of the dispersal ability of ‘genetically sterile’ male *Ae*. *aegypti* mosquitoes using data from large-scale MRR experiments carried out at an urban field site in Brazil. The analysis facilitates the quantification of dispersal through the parameterisation of a dispersal kernel for the released insects. Many summary measures of interest relating to dispersal may be drawn from such a kernel. To enable a comparison of both the biological outcomes and methodology employed, the analytical methods are also used to re-analyse published data on the dispersal of ‘genetically sterile’ male *Ae*. *aegypti* at an uninhabited forested site in Pahang, Malaysia [12]. The dispersal ability of the ‘genetically sterile’ insects was previously analysed in the Malaysian study using methods detailed in Morris et al. (1991) [13] and evaluated the MDT to be 52.4m (95% CI: 41.6m, 61.4m) [12]. The aims of the re-assessment of dispersal ability are: i) to assess the robustness of the estimate of dispersal from Brazil data to habitat and locational heterogeneities, ii) to explore potential differences in dispersal behaviour between sites and iii) to assess the applicability of the dispersal kernel method in comparison with more common approaches to estimating and quantifying dispersal.

## Methods

### Ethics statement

Before establishment of the ‘genetically sterile’ male *Ae aegypti* line in the mass rearing laboratory and subsequent open releases, regulatory approvals were obtained from the appropriate Brazilian national regulatory body: the Brazilian National Biosafety Technical Commission (CTNBio). Releases were preceded by community engagement with consent and support obtained from regional (Bahia health secretary) and local community leaders (Town Mayor, health secretary and vector control authorities). Prior to sampling, informed consent was received from the landowners.

### Study site

**Fig 1:**
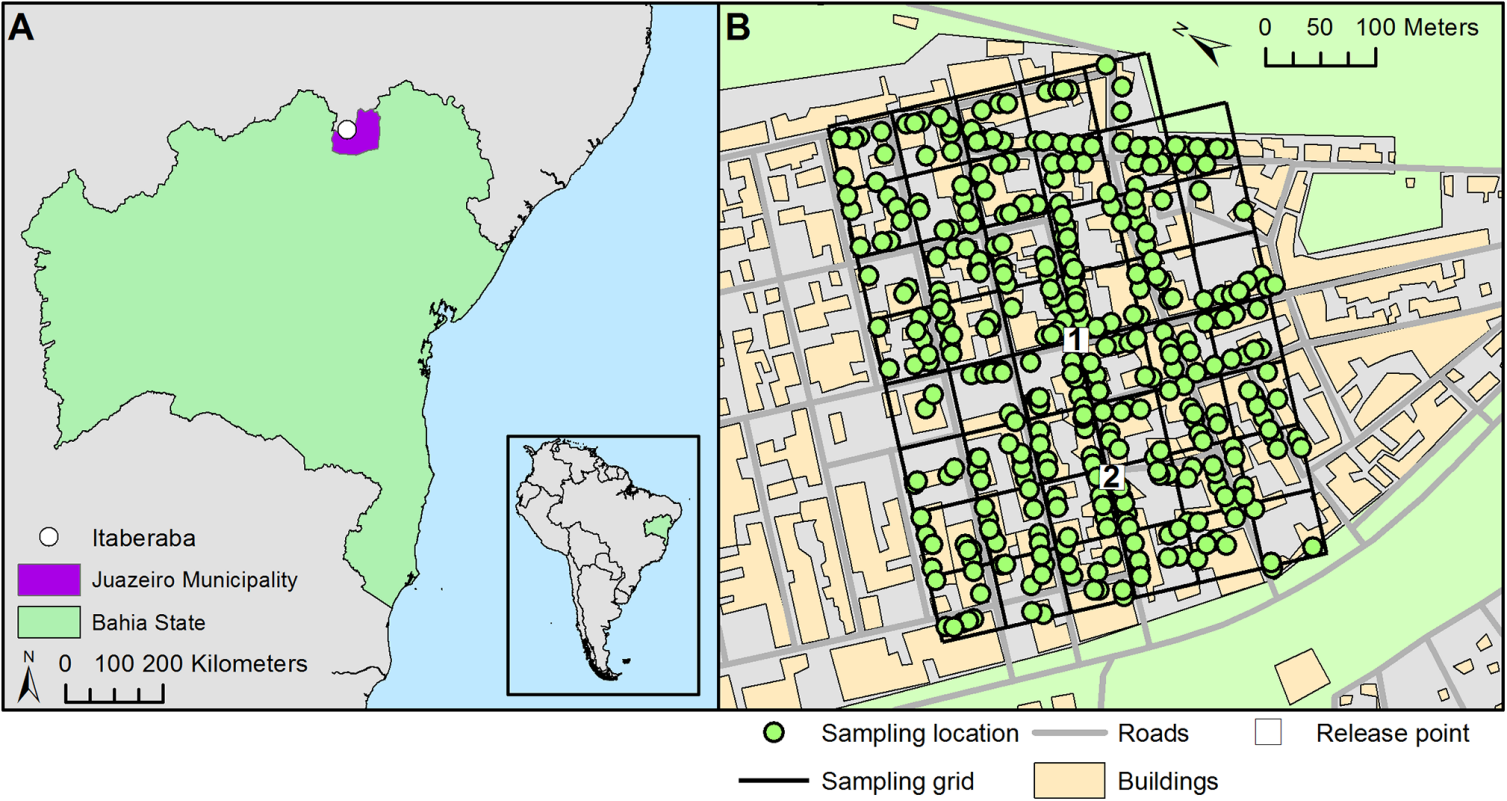
Brazil study site sampling locations. A) The study site, Itaberaba, a suburb of the city of Juazeiro, Bahia State, Brazil. B) MRR release points (numbered squares) and sampling locations (green circles) within households distributed across the sampling grid at the study site.

The field site is located in Itaberaba, a suburb of the city of Juazeiro, Bahia, Brazil (Latitude: -9° 26' 59", Longitude: -40° 28' 53") (Fig 1A). The site is located in a semi-arid part of Brazil and consists mainly of low-socioeconomic status residential housing. The majority of houses at the study site were single-story brick and concrete buildings with unscreened windows. The habitat across the sampled region was a homogenous urban environment.

### Mark-release-recapture

The ‘genetically sterile’ line used was OX513A and was reared according to methodologies given in Carvalho *et al*. (2014) [19].

A total of 19,164 ‘genetically sterile’ male *Ae*. *aegypti* formed three releases. Individuals from each release were marked with the same coloured fluorescent powder (www.dayglo.com). Release 1 (red release) and release 2 (blue release) were performed on 21 February 2011 and consisted of 5,349 and 5,465 individuals released from points 1 and 2 (Fig 1B) respectively. Release 3 (yellow release) was performed on 25 February 2011 with 8,350 individuals released from point 1 (Fig 1B).

Aspiration sampling was used to recapture marked adults. Sampling was conducted using locally made battery powered hand-held aspirators. After obtaining consent from the respective property owner, each building was sampled for a set period of 15 minutes. Sampling locations were distributed across the study site (Fig 1B) and sampling was conducted for up to nine days post release (S1 Data). Sampling locations were chosen by randomly selecting one household from each of the 47 (60m by 60m) grid squares each day (with the exception of day 5 for the red and blue release and day 1 for the yellow release where, for logistical reasons, the number of households sampled was lower). Each mosquito collected was assessed to determine the i) origin (‘genetically sterile’ or wild as indicated by the presence/absence of fluorescent powder respectively), ii) sex and iii) genus (*Aedes* or non-*Aedes*). Weather variables (daily maximum temperature and maximum humidity) were recorded from a local weather station (situated approximately 10.2km north-west of Itaberaba).

A secondary analysis of the MRR data from a previously published study in Malaysia [12] was undertaken to obtain a comparative second estimate of the ‘genetically sterile’ insect’s dispersal ability and associated density kernel. In this study a total of 6,045 marked ‘genetically sterile’ males were released at a forested site in Pahang, Malaysia. For a detailed description of the study site and MRR methods please see Lacroix *et al*. (2012) [12].

### Model framework

All multivariable analyses were performed within a GLM framework. In instances where the number of recaptures is small relative to the total releases, the Poisson regression model may be used as an appropriate approximation [20].

We assume that the count response variable (recaptures) is Poisson distributed with mean *μ* and variance *μ*

$$Y_{i}∼Pois(\mu_{i}).$$ (1)

The response must be ≥ 0. Therefore a log link function is used to link the mean to the explanatory variables

$$\text{ln}\left(\mu_{i}\right)=x_{i}\beta ,$$ (2)

where *x*_*i*_ *β* is a linear predictor

$$x_{i}\beta =\beta_{0}+\beta_{1}x_{i1}+\ldots +\beta_{p}x_{ip},$$ (3)

where *β* denotes the unknown parameters to be estimated and *x*_*i*_, the explanatory variables. Parameter estimates were obtained by maximising the log-likelihood (l) of the data:

$$\begin{array}{l} l\left(\beta |Y\right)=\sum_{i=1}^{n}\left(y_{i}\;\text{ln}\;\mu_{i}-\mu_{i}-\text{ln}(y!)\right)\\ \;=\sum_{i=1}^{n}\left(y_{i}x_{i}\beta -\text{exp}\left(x_{i}\beta \right)-\text{ln}\;y_{i}!\right). \end{array}$$ (4)

In the situation where the response variable is overdispersed (variance>>mean) a Poisson GLM, where the variance is assumed to equal the mean, would be misspecified. In this instance, the negative binomial GLM, detailed below, may be used [21–23].

We assume that the count response variable follows a negative binomial distribution. A Poisson model is used for the count, conditional on the mean value, *Z*_*i*_, which is assumed to have a gamma distribution, with mean, *μ*_*i*_, and constant scale parameter, *θ*

$$Y_{i}∼Poisson\left(Z_{i}\right),\;Z_{i}∼gamma(\mu_{i},\theta ).$$ (5)

Therefore the expected value of *Y* and the variance of *Y* are as follows

$$E\left(Y_{i}\right)=\mu_{i},\;Var\left(Y_{i}\right)=\mu_{i}+\frac{\mu_{i}^{\;2}}{\theta }$$ (6)

The mean response, *μ*_*i*_, may be linked to a linear combination of explanatory variables using the log link function (Eq (2)) and linear predictor (Eq (3)). Parameters were estimated by maximising the log-likelihood (l) of the model:

$$l(\beta ,\theta |Y)=\sum_{i=1}^{i=n}\theta (\text{ln}(\theta )-\text{ln}(\theta +\mu_{i}))+\text{ln}(\Gamma (\theta +y_{i}))-\text{ln}(y_{i}!\Gamma (\theta ))-y_{i}(\text{ln}(\mu_{i})-\text{ln}(\theta +\mu_{i})),$$ (7)

where the gamma function, Γ, is

Γ(n)=(n−1)! (8)

### Dispersal kernels

Considerable inconsistencies abound regarding different interpretations of the term ‘dispersal kernel’ [18,24,25]. Two kernel definitions, often used interchangeably, are i) the probability density function (pdf) of the dispersal distance of each disperser. Referred to as the distance kernel [18] or the distance pdf [25] and ii) the density of probability of a given bearing and dispersal distance from the source. Referred to as the location kernel [18] or the density pdf [25].

**Fig 2:**
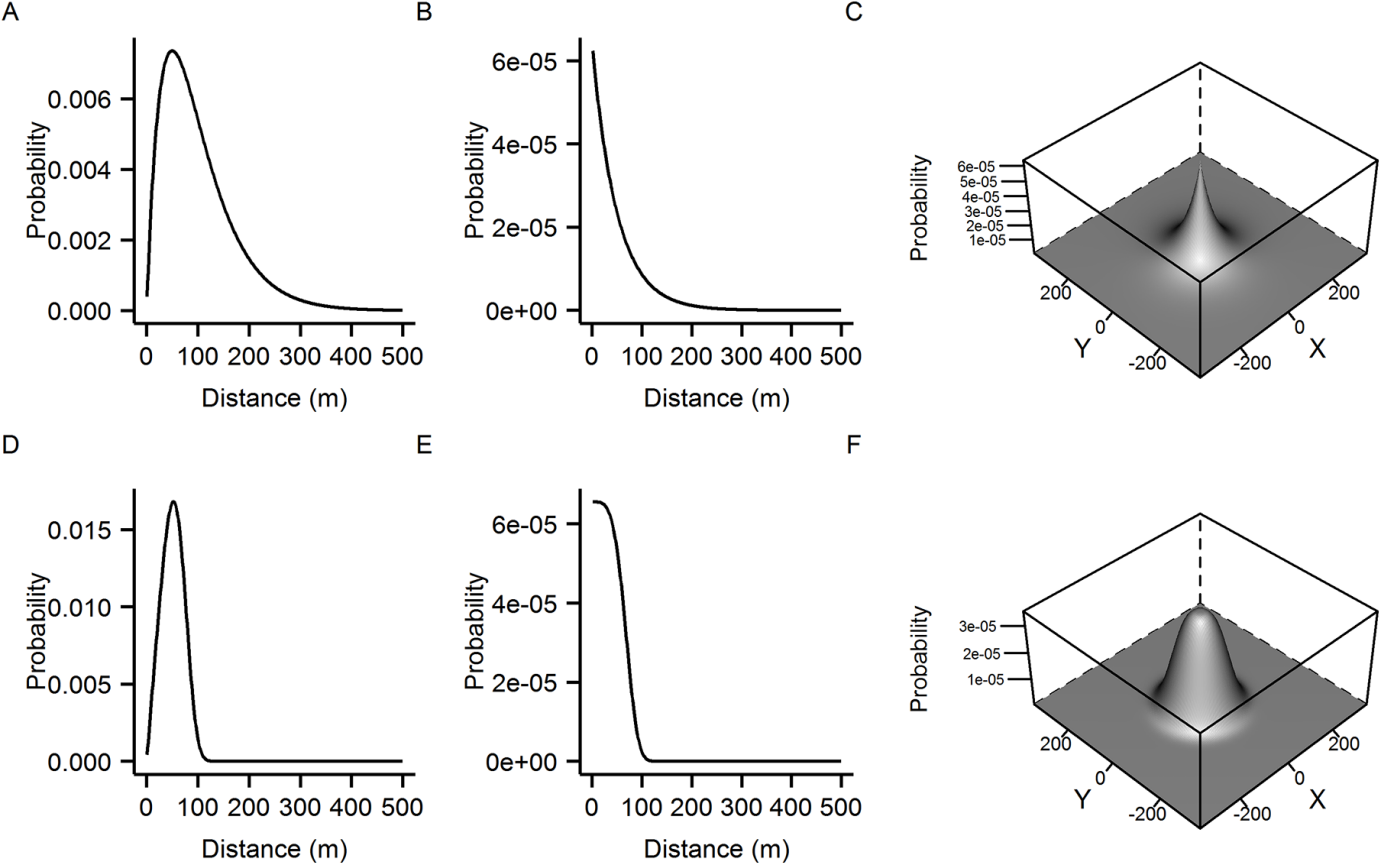
Dispersal kernels. Examples of different kernel interpretations for the negative exponential (A, B and C) and exponential power (D, E and F) kernels. The distance pdf is shown in panels A and D. The density with respect to distance is shown in panels B and E and the density pdf is illustrated in panels C and F (after Cousens *et al*. [25]). Kernels in A, D, C and F integrate to unity (in 1 dimension for the distance pdfs and 2 dimensions for the density pdfs).

We adopt the terminology of Cousens *et al*. [25], henceforth referring to kernel type 1 as the distance pdf and type 2 as the density pdf. Both kernel types are true pdfs, integrating to 1 (the density pdf is integrated over the whole 2d space). Both kernel types are closely related. The distance pdf can be derived by multiplying the density pdf by *2πd* where *d* is the distance from the source [18] (assuming radial symmetry). Examples of these kernel types are shown in Fig 2.

The density pdfs, assuming radial symmetry, used in this analysis are defined by the following functions

$$Negative\;exponential\;kernel=\frac{1}{2\pi a^{2}}{\text{e}}^{\left(-\frac{d}{a}\right)}\;a>0,$$ (9)

$$Exponential\;power\;kernel=\frac{b}{2\pi a^{2}\Gamma \frac{2}{b}}e^{\left(-\frac{d^{b}}{a^{b}}\right)}\;a,b>0,$$ (10)

where *d* is the distance (metres), *a* and *b* are kernel parameters and Γ the gamma function (Eq (8)) [18]. The negative exponential kernel is characterised by the exponential power kernel when *b* = 1. The associated MDT functions are

NegativeexponentialMDT=2a (11)

$$Exponential\;power\;MDT=a\left(\frac{\Gamma \frac{3}{b}}{\Gamma \frac{2}{b}}\right).$$ (12)

Estimates of FR_50_ and FR_90_ are made by assessing the cumulative distribution of the distance pdf at the 50% and 90% levels.

### Variables

The outcome variable was the number (count) of marked ‘genetically sterile’ male *Ae*. *aegypti* recaptured. Potential explanatory variables included in the Brazil analysis were: i) a spatial measure which could either be the measured distance (m) between release and recapture or the density as calculated by a parameterisation of a given density pdf, ii) the number of days post release, the effect of which is assumed to be linear, iii) the number of wild *Aedes* species collected iv) the number of non-*Aedes* wild mosquitoes collected, v) the maximum temperature (°C) on the day of collection, vi) the maximum humidity (%) on the day of collection and vii) the directional quadrant, North, South, East or West (relative to release point) that the collection was made in.

Due to the relatively low recapture number, data from all three MRR experiments were combined for analysis.

For the Malaysia analysis the outcome variable was the number (count) of marked ‘genetically sterile’ male *Ae*. *aegypti* recaptured. Potential explanatory variables included in the analysis were: i) a spatial measure which could either be the measured distance (m) between release and recapture or the density as calculated by a parameterisation of a given density pdf, ii) the number of days post release, the effect of which is assumed to be linear iii) the number of wild *Aedes* species (specifically: *aegypti*, *albopictus* and *togoi*) collected, iv) the number of wild *Culex* collected and v) a categorical variable indicating if the recapture location was uphill or downhill from the release site.

Three models were evaluated to compare different transformations of the distance explanatory variable. Model 1 incorporated all explanatory variable including distance, Model 2 all explanatory variables including distance density (negative exponential kernel) and model 3 all explanatory variable including distance density (exponential power kernel).

For model 1 the full model was fitted using maximum likelihood techniques, utilising the GLM and Negative binomial GLM function of the statistical software package R [26] with the MASS package [21]. All explanatory variables were included in the initial model as well as an interaction term between distance and day post release. Model selection by minimising the Akaike information criterion (AIC), a penalised likelihood score, was then performed using the MASS package [21]. The AIC is calculated as

AIC=2k−2ln(L) (13)

where *k* is the number of parameters and L the maximised likelihood value.

For models 2 and 3 fitting was performed using the following process. First, the distance density was estimated using the assigned kernel. The GLM was then fitted using the same process as for model 1, as a function of the transformed distance explanatory variable. These steps were then optimised over the kernel parameter space allowing identification of the optimal combination of explanatory variables and kernel parameters as indicated by the AIC. The best overall model was judged to be the one with the minimum AIC value.

For comparison, the estimated survival of released insects in the Brazil study predicted using the GLM was also calculated using a non-linear regression approach [27], that was also used in the original analysis of the Malaysia data [12].

### Kernel confidence intervals

Following model estimation, 95% confidence intervals were calculated for the maximum likelihood kernel parameter estimates using the profile likelihood method. The maximised log-likelihood with respect to *β*, *α* and *b*, i.e. that corresponding to the maximum likelihood estimates (MLEs) of *β*, *α* and *b*, is defined as $l(\hat{\beta },\hat{a},\hat{b}|Y)$.

First kernel parameter *a* was increased or decreased in small increments whilst *β* was held at the MLE $\left(\hat{\beta }\right)$ and kernel parameter *b* was optimized conditional on $\hat{\beta }$ and the assumed value of *α* (*a*_0_), giving the log-likelihood:

$$l\left(\hat{\beta },a_{0},\tilde{b}|Y\right),$$ (14)

where $\tilde{b}=MLE(b|a_{0},\hat{\beta })$.

Secondly, kernel parameter *b* was increased or decreased in small increments whilst *β* was held at the MLE ($\hat{\beta }$) and kernel parameter *α* was optimized conditional on $\hat{\beta }$ and the assumed value of *b*(*b*_0_), giving the log-likelihood:

$$l\left(\hat{\beta },\tilde{a},b_{0}|Y\right),$$ (15)

where $\tilde{a}=MLE(a|b_{0},\hat{\beta })$.

After each change the log-likelihood of the model was recalculated and a corresponding test statistic assessed. For example, evaluating for *a*, the *G* ^*2*^ statistic was calculated

$$G^{2}=2\left(l(\hat{\beta },\hat{a},\hat{b}|Y)-l\left(\hat{\beta },a_{0},\tilde{b}|Y\right)\right).$$ (16)

The *G* ^*2*^ statistic was compared to the χ^2^ distribution (with 1 degree of freedom) for the (1-α) percentile. Thus for 95% confidence intervals the critical *G* ^*2*^ value is 3.84. The log-likelihood surface was calculated with respect to kernel parameters of the optimal model for exploration and visualisation of the parameter space for both the Brazil and Malaysia analyses.

## Results

### Primary analysis—Brazil

**Fig 3:**
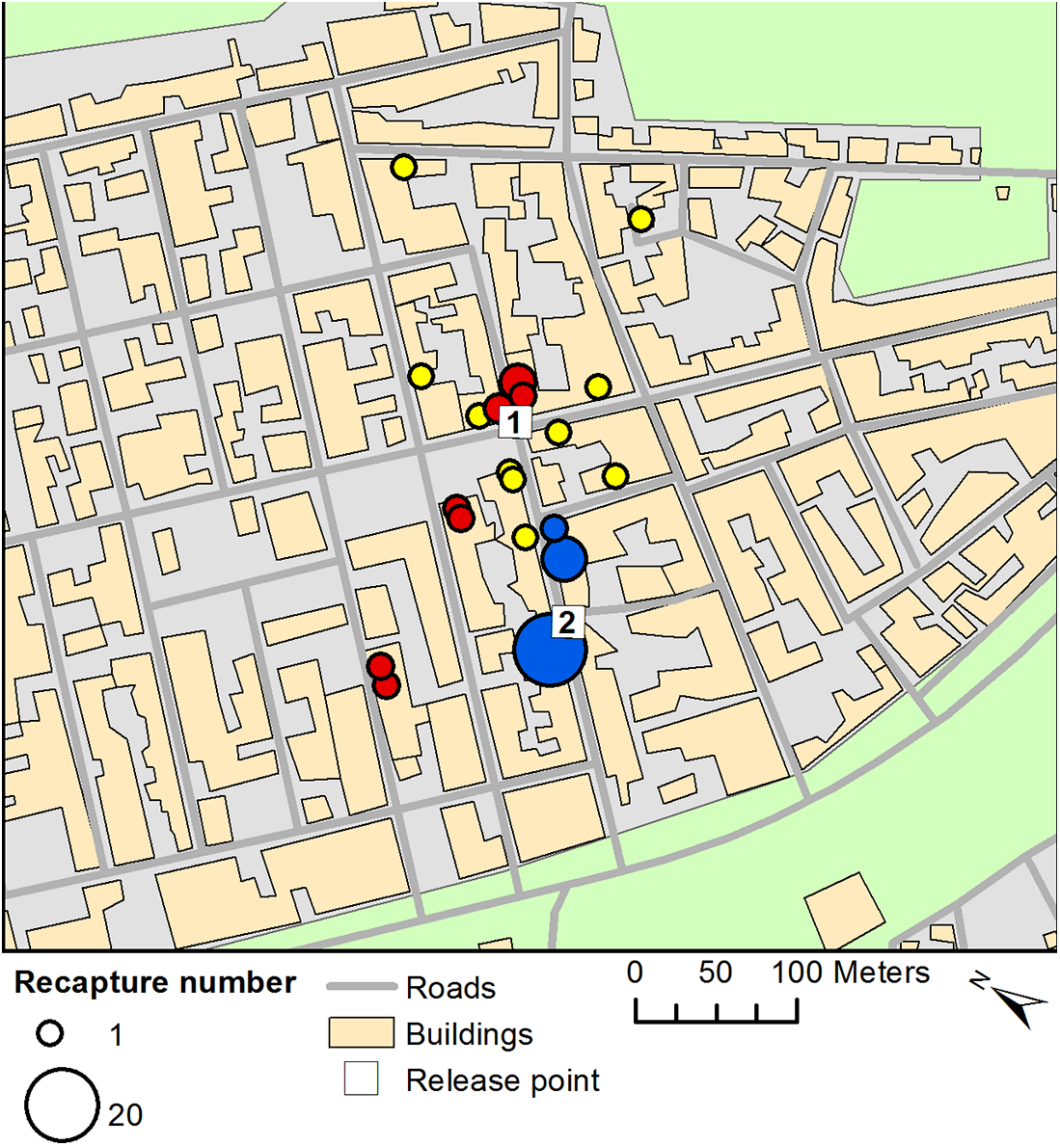
Recaptures for three MRR experiments at the field site in Brazil. Numbered squares represent the two release points for MRR experiments. Coloured circles indicate the location and size of recaptures for three separate MRR releases (insects marked with red, yellow and blue fluorescent powder).

Recaptures for the three MRR experiments are summarised in Table 1. The locations of recaptures are shown in Fig 3. The mean count of recaptured marked males (the response), per sample, per day was 0.077 (variance = 0.73). Over the recapture period the maximum daily temperature ranged between 25.4°C-34.6°C and the maximum relative humidity between 66%-92%.

**Table 1:** Summary data of the three MRR experiments in Brazil.

Release	Release date	Number released	Release point	Number (%) recaptured
Red	21-Feb-2011	5,349	1	22 (0.4)
Yellow	25-Feb-2011	8,350	1	17 (0.2)
Blue	21-Feb-2011	5,465	2	30 (0.5)
**Total**	-	19,164	-	69 (0.36)

**Fig 4:**
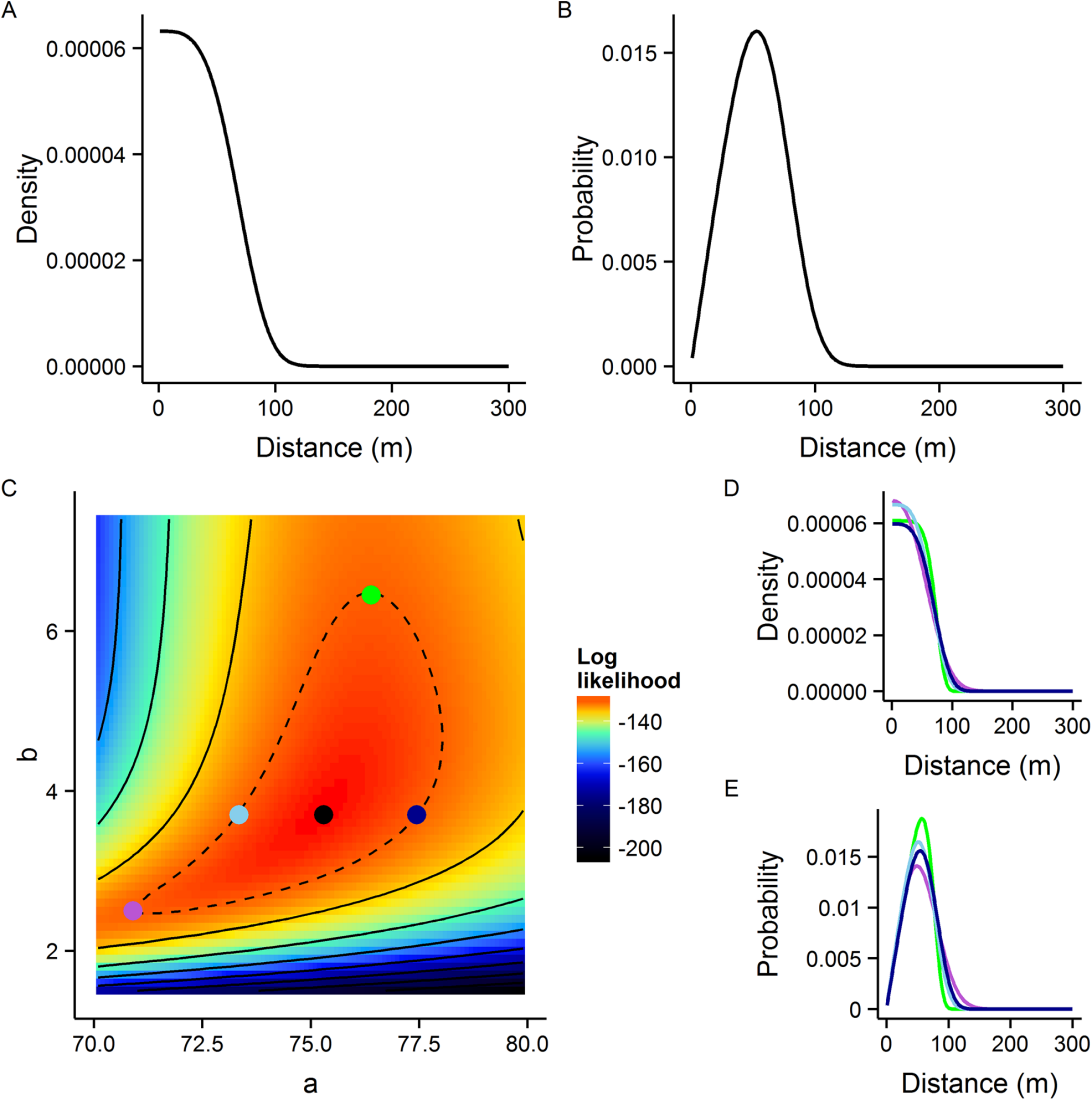
Dispersal kernel summary for the Brazil analysis. A) Maximum likelihood estimate of density with respect to distance for Brazil data. B) Maximum likelihood distance pdf. C) The log-likelihood surface with respect to kernel parameters *a* and *b*, coloured points highlight the MLE (black, log-likelihood = -128.1) and examples of extreme 95% CI (green, light blue, dark blue and mauve) kernel parameter combinations. The dotted line demarks the 95% confidence interval contour. Solid black contour lines are at intervals of 10 log-likelihood. Examples of D) distance densities and E) distance pdfs from the 95% confidence interval range corresponding to the coloured points shown in panel C.

A summary of model performance using the untransformed- and transformed-distance explanatory variable is shown in Table 2. Combining all available data (from the red, yellow and blue releases) the best fitting model (lowest AIC) incorporated the exponential power kernel. The maximum likelihood exponential power density pdf has an associated MDT of 52.8m (95% CI: 49.9m, 56.8m), FR_50_ of 52.4m (95% CI: 50.6m, 54.7m) and FR_90_ of 83.0m (95% CI: 74.8m, 93.9m). The MLE kernel, log-likelihood surface and examples of kernels drawn from 95% CI parameter values are shown in Fig 4.

**Table 2:** Brazil model performance and kernel parameter estimates.

Distance transformation	AIC	Explained variance (%)	Number of covariates	Kernel parameter estimates (95% CI)	
				*a*	*b*
**Untransformed**	293.5	46.7	5	-	-
**Negative exponential**	286.5	48.6	6	97.8 (57.8,238.3)	-
**Exponential power**	**276.2**	**51.4**	**5**	**75.3 (66.1,85.0)**	**3.7 (2.0,7.3)**

The estimated model performance (minimum AIC indicated in bold) and kernel parameters for different transformations of the distance explanatory variable for the Brazil analysis.

For all three models the distance or distance density and the number of days post release were strongly associated with recapture number. There was no evidence of an interaction between distance (untransformed or transformed) and the number of days post release in any of the models considered. Assuming no emigration, the estimated mortality rate of released insects of 0.62 (95% profile likelihood CI: 0.84, 0.42) would equate to a mean average lifespan of 0.62^−1^ = 1.61 days (95% CI: 1.19 days, 2.38 days). Estimates of the mean average lifespan calculated using the non-linear regression approach [27] were 1.00 days (95% bootstrapped CI: 0.63 days, 1.48 days).

Other significant explanatory variables were the number of non-*Aedes* mosquitoes recorded from the sample and the maximum humidity. There was evidence of a lack of radial symmetry in dispersal from the release point as the quadrant explanatory variable was also associated with recapture number. A summary of the parameter estimates from the optimal model, using the exponential power transformation of distance as an explanatory variable is shown in Table 3.

**Table 3:** Brazil model coefficient estimates.

Coefficient		Estimate	Standard error	z-value	p-value
**Intercept**		-1.35	1.18	-1.16	0.25
**Transformed distance**		83,140	7,015	11.85	<0.0001
**Number of days post release**		-0.62	0.11	-5.84	<0.0001
**Wild other spp**		0.021	0.0079	2.62	0.0088
**Maximum humidity**		-0.035	0.016	-2.22	0.027
**Quadrant** *	North	1	-	-	-
	South	-1.88	0.80	-2.36	0.018
	East	0.76	0.37	2.07	0.039
	West	1.40	0.35	3.97	0.00071

GLM coefficient estimates and associated standard errors, z-value and p-values from the optimal model for the Brazil analysis. Distance was transformed using the exponential power kernel.

*Overall significance level p<0.0001 (χ^2^ = 46.50, 3df).

### Secondary analysis—Malaysia

The MRR performed in Malaysia was associated with consistently higher recaptures than the MRR experiments in Brazil. Of 6,045 released ‘genetically sterile’ males 3,034 (50.2%) were recaptured over the 15-day course of the experiment. The count of recaptured, marked males (the response) was very overdispersed (mean = 2.6 per sample per day, variance = 523), and therefore a negative binomial GLM was fitted. A summary of the model performance using the untransformed and transformed distance explanatory variable is shown in Table 4.

**Table 4:** Malaysia model performance and kernel parameter estimates.

Model	AIC	Explained variance (%)	Number of covariates*	Kernel parameter estimates (95% CI)	
				a	b
**Untransformed**	728.7	7.9	3	-	-
**Negative exponential**	668.4	54.1	3	31.3 (27.7,34.9)	-
**Exponential power**	**668.0**	**46.8**	**3**	**48.1 (45.3, 52.1)**	**1.4 (1.3,1.5)**

The estimated model performance (minimum AIC indicated in bold) and kernel parameters for different transformations of the distance explanatory variable for the Malaysia analysis.

*Including the interaction term.

**Fig 5:**
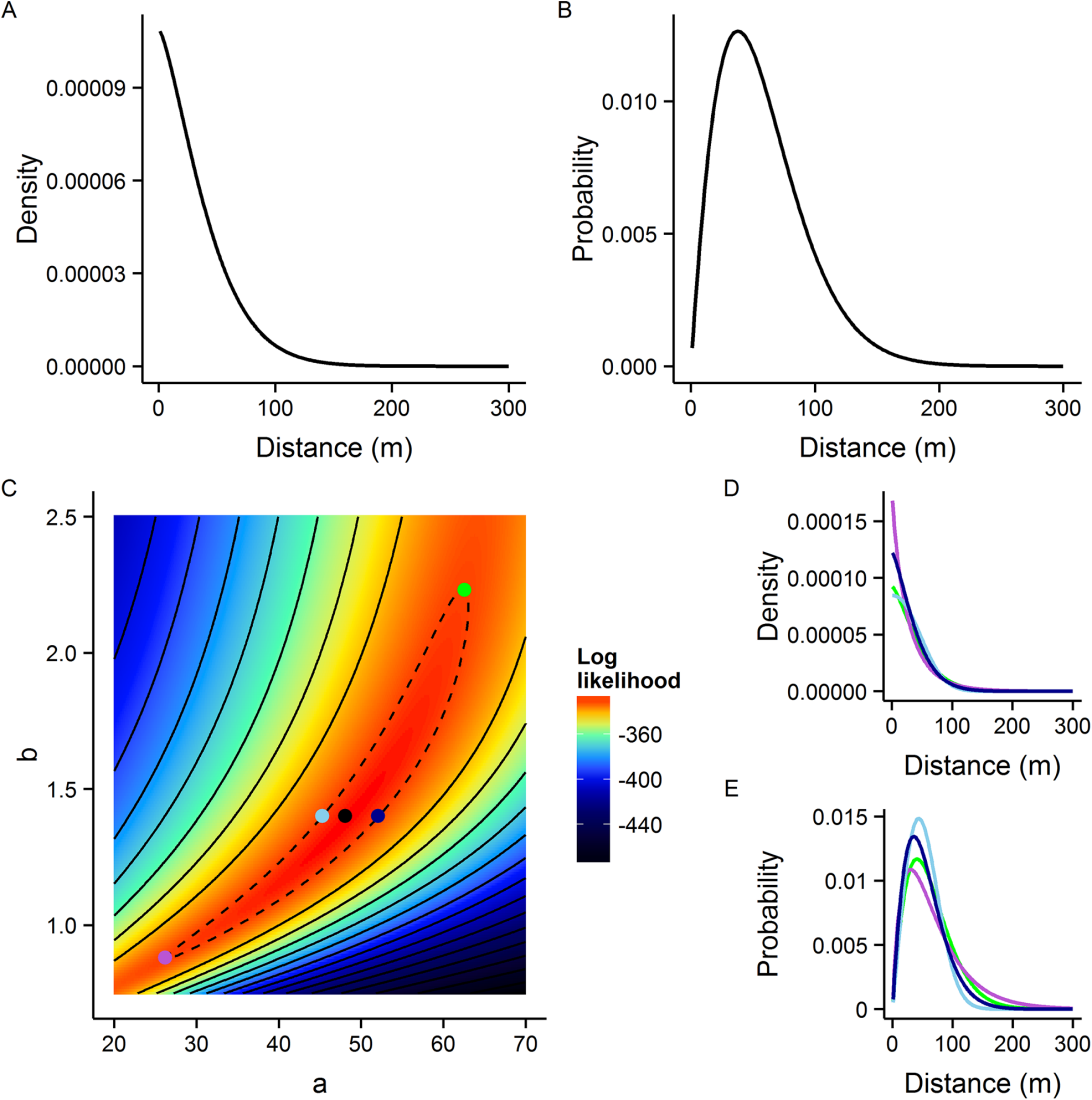
Dispersal kernel summary for the Malaysian analysis. A) Maximum likelihood estimate density with respect to distance for Malaysian data. B) Maximum likelihood distance pdf. C) The log-likelihood surface with respect to kernel parameters *a* and *b*, coloured points highlight the MLE (black, log-likelihood = -328) and examples of extreme 95% CI (green, light blue, dark blue and mauve) kernel parameter combinations. The dotted line demarks the 95% confidence interval contour. Solid black contour lines are at intervals of 10 log-likelihood. Examples of D) distance densities and E) distance pdf from the 95% confidence interval range corresponding to the coloured points shown in panel C.

Again, the optimal model, as determined by AIC, used the exponential power density pdf, although the negative exponential density pdf produced only marginally inferior fit (AIC = 668.0 and 668.4 for the exponential power and negative exponential kernels respectively). The MLE exponential power pdf estimates a MDT for the ‘genetically sterile’ release of 58.0m (95% CI: 51.1m, 71.0m), FR_50_ of 51.8m (95% CI: 47.9m, 58.7m) and FR_90_ of 105.7m (95% CI: 86.5m, 141.1m). The MLE kernel, log-likelihood surface and examples of kernels drawn from 95% CI parameter values are shown in Fig 5.

The coefficient summaries from the negative binomial model using the exponential power transformed distance explanatory variable are shown in Table 5. This second analysis again indicates that distance is an important significant predictor of the expected count of recaptures. The number of days post release was also significantly associated with recapture number. Assuming no emigration, the estimated mortality rate of released insects of 0.46 (95% profile likelihood CI: 1.03, 0.13) would equate to a mean average lifespan of 0.46^−1^ = 2.17 days (95% CI: 0.97 days, 8.85 days). Unlike the analysis for Brazil there was evidence of an association between the distance and the number of days post release explanatory variables.

**Table 5:** Malaysia model coefficient estimates.

Coefficient	Estimate	Standard error	t-value	p-value
**Intercept**	-0.013	0.79	-0.016	0.98
**Transformed distance**	96,300	17,500	5.50	<0.0001
**Number of days post release**	-0.46	0.22	-2.10	0.036
**Interaction (Transformed distance × Days post release)**	-24420	6467	-3.78	0.0002

GLM coefficient estimates and associated standard errors, t-values and p-values from the optimal model for the Malaysia analysis. Distance was transformed using the exponential power kernel.

**Fig 6:**
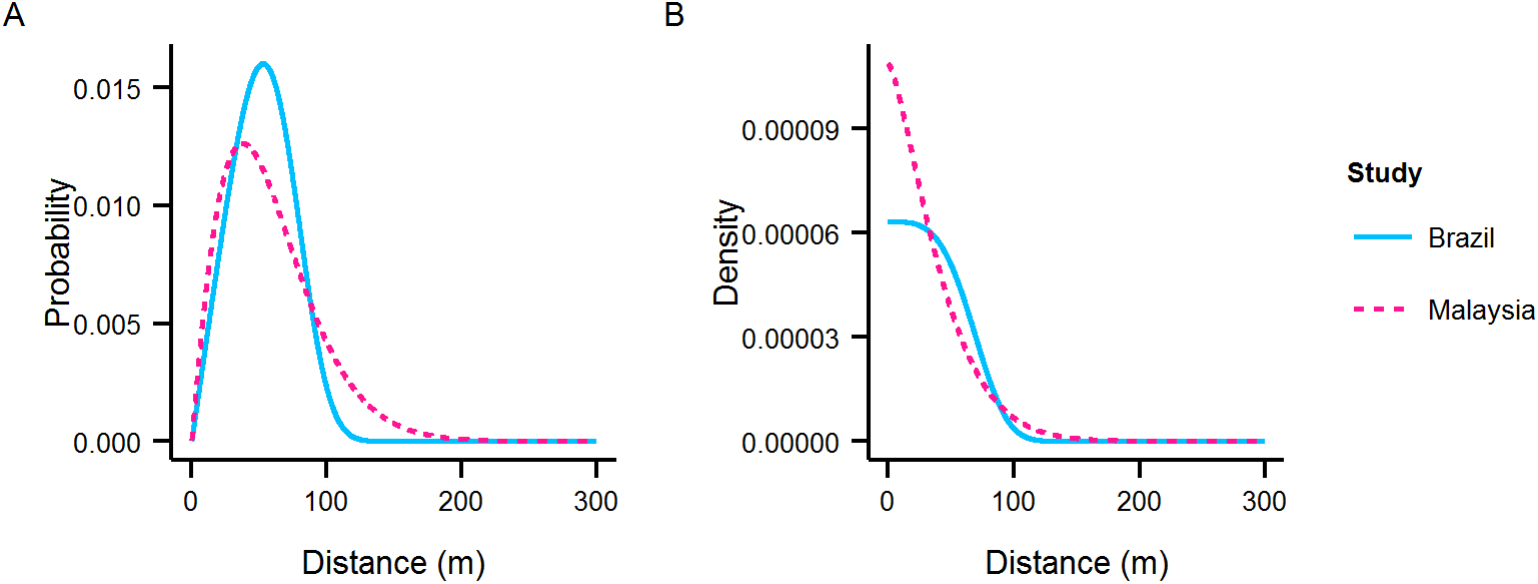
Dispersal kernel comparison. A comparison of the A) distance pdf and B) density with respect to distance for estimates using MRR data from Brazil (solid blue line) and Malaysia (dashed pink line). The comparison highlights the similarity in estimated kernels for experiments conducted on different continents, in different habitats.

For a direct comparison the distance pdf and density with respect to distance for the optimal kernels estimated from the Itaberaba and Malaysia MRR experiments have been overlaid (Fig 6).

## Discussion

An in-depth analysis of the dispersal ability of released ‘genetically sterile’ male *Ae*. *aegypti* mosquitoes in the field has been conducted. The primary analysis, of MRR data from Brazil, indicates distance from the release point to be an important predictor of the expected number of recaptures. The relationship between the recapture number and the distance from the release point is highly non-linear. The regression model performed optimally when an exponential power dispersal kernel was used to transform distances. The analysis methodology was also used to re-analyse MRR from Malaysia, where again an exponential power kernel provided best model fit.

The optimal Brazil GLM performed well, explaining around half of the variation observed in the data. Transformed distance and the number of days post release were the most influential, highly significant predictors of recapture number. The decline in numbers temporally after release is considered to be predominantly due to the effect of mortality. The effect of emigration from the study area can be important [28] and would further reduce numbers but was assumed to be small due to the size of the study area and low recaptures at the periphery.

Distance was highly significantly correlated with recapture number in all models considered. The exponential power dispersal kernel provided the optimum model fit. This kernel parametric form is slightly more flexible than the negative exponential. The kernel produced showed a high and consistent level of dispersal from 0-33m from the release site. After this point the density falls fairly steeply, reaching very low levels shortly after 100m (FR_90_ = 83.0m), indicating that coverage decreases quickly at increasing distances more than 33m from a release point. The MDT estimated using the best-fit kernel parameters was 52.8m (95% CI: 49.9m, 56.8m). This is consistent with a number of published field studies of male *Ae*. *aegypti* dispersal which estimate mean distance travelled to range from 10m to 100m (Table 6). There are however, a limited number of studies of male *Ae*. *aegypti* dispersal as focus has been on the biting females. It is however, important to note that for skewed distributions of dispersal distances the MDT as a measure of central tendency should be interpreted with some caution.

**Table 6:** Summary of a literature review of male *Ae*. *aegypti* dispersal estimates.

MDT/MDT range (m)	Location	Notes	Reference
10–30	Hainan Island, China	Released in the centre of a village	[8]
15–39	Sonepat, India	-	[9]
32	Ilha do Governador, Brazil	Raised on poor diet	[10]
35	Pentland, Australia	-	[11]
35–60	Hainan Island, China	Released at the edge of a village	[8]
42	Ilha do Governador, Brazil	Raised on rich diet	[10]
52	Jalan Tentera, Malaysia	Transgenic	[12]
100	Jalan Tentera, Malaysia	Laboratory strain	[12]

The number of non-*Aedes* mosquitoes was significantly positively correlated with the number of recaptured ‘genetically sterile’ mosquitoes. This explanatory variable is thought to be a proxy for the house-attractiveness or accessibility of a house to mosquitoes; large numbers of other mosquito species may indicate that the household is situated in a favourable location or particularly amenable or attractive to mosquitoes. Clustering of *Aedes* mosquitoes at the household level is a commonly observed phenomenon [29–31]. An alternative explanation could also lie in differences in the abilities of operators to sample mosquitoes.

The humidity and directional quadrants were significantly associated with the number of recaptured ‘genetically sterile’ mosquitoes but had small effect sizes. It would be expected that any directional differences are attributable to site- and time-specific heterogeneities in terrain, habitat type, wind direction or other external factors [32–34]. Humidity was positively associated with recapture number, which could be due to an increased tendency for *Ae*. *aegypti* to seek shelter with increasing humidity [35]. These explanatory variables must be interpreted with some caution as there is the potential for selection by AIC to overfit models [36,37]. The covariates may therefore be included in the final models despite their relatively small influence on model fit.

One limitation of this study was the low number of recaptures in the Brazil dataset. For this reason there was little power to analyse individual releases separately, necessitating the analysis of the combined datasets and the assumption that influences not accounted for would be similar across all three releases. The proportion of individuals recaptured may have been improved by a more targeted, or higher intensity, sampling effort or increased survival of the released individuals. Alternatively, the absolute number of recaptures could have been increased with larger release numbers. We have assumed throughout that aspiration will not remove all fluorescent dust from the marked individuals [38]. The dataset could also have been further improved with household-specific monitoring of climatic factors to give greater resolution to observations on the relationship between meteorological variables and recapture numbers. The standard errors of the GLM coefficients (Tables 3 and 5) were estimated conditional upon the MLEs for kernel parameters *a* and *b* whilst the confidence intervals for the kernel parameters were obtained conditional upon the MLE for the GLM coefficients. Thus the reported standard errors are likely to be smaller than if we had been able to compute the unconditional standard errors and confidence intervals for all of the parameters. Analysis of the residuals indicated little residual spatial autocorrelation with perhaps the exception of some under-estimation of recapture numbers at further distances (>150 m), potentially indicating the influence of long-distance dispersers [39], although, due to the small number of recaptures, this is difficult to verify.

The optimal GLM associated with the Malaysia data also explained approximately 50% of the variation observed in the recapture data. For the optimal model only two covariates, number of days post release and distance, plus their interaction term were included. The optimal model again used the exponential power dispersal kernel, with a corresponding MDT estimate of 58m (95% CI: 51.1m, 71.0m) that corroborated the MDT estimate of 52.4m (95% CI: 41.6m, 61.4m) from the previously published analysis of these data [12]. The FR_50_ estimate of 51.8m (95% CI: 47.9m, 58.7m) was substantially different from the previously published estimates of 16.2m (95% CI: 10.5m, 22.5m), a product of the different underlying models for dispersal with respect to distance used in each analysis. This deviation further highlights the potential benefits of more accurately characterising dispersal behaviour. The number of days post release covariate, as expected, was significantly negatively associated with recapture number. The associated average lifespan of 2.17 days (95% CI: 0.97 days, 8.85 days) was in good agreement with the previously published estimate of 2 days (95% CI: 1.8 days, 2.2 days). The coefficient indicated a smaller effect size than seen in the Brazil MRR data, implying improved survival, less emigration or a combination of the two for individuals in the Malaysia releases. The recapture rate in the Malaysia MRR experiment was very high, approximately 50% of all individuals released were recaptured. This may bias the results and could violate the underlying assumption that the negative binomial distribution approximates proportions when recapture numbers are small relative to the release size.

The lack of significant interaction between the number of days post release and distance in the Brazil data provides evidence for a single main dispersal event on release (the probability of travelling a given distance is not influenced by the number of days post-release). In the Malaysia analysis the significant interaction term indicates a more continual dispersal process over time, possibly due to the lack of favourable (urban/peri-urban) habitat across the whole range of the study site at this location. However the influence of the interaction term on predicted recaptures is very small; the majority of released individuals have died (or emigrated) before the interaction term becomes influential. For both locations the majority of recaptures are predicted spatially and temporally close to the release location and date respectively. There is published evidence to support either the occurrence of a single dispersal event [40–42] or a more continuous dispersal process upon release [8,9,11].

For experiments carried out in different habitats, on different continents, the estimated dispersal kernels were very similar (Fig 6). The Brazil dispersal kernel is slightly fatter tailed (larger *b* parameter) but in general there is evidence for a degree of consistency in the dispersal ability of ‘genetically sterile’ male *Ae*. *aegypti* across a range of environments. Consistent dispersal may facilitate more generalised release procedures for sterile insect releases across a range of release locations and scenarios.

Accurately measuring and assessing the dispersal of released ‘genetically sterile’ male *Ae*. *aegypti* in the field is a vital component necessary to optimise vector control using these genetically sterile individuals. A successful control program using ‘genetically sterile’ male *Ae*. *aegypti* would maximise ‘genetically sterile’ insect density over the target area. Knowledge of the released insects’ ability to disperse is vital in predicting their density with respect to specific release points or routes. An ability to predict the coverage of dispersed individuals will facilitate the design and implementation of more efficient control and monitoring programs in the future.

## Supporting Information

S1 Data Brazil MRR data. Georeferenced recapture number with respect to release, time and meteorological variables.
(TXT)
